# Hydrogen‐Bond Enabled Phosphite Based Intrinsic Flame‐Retardant Solid‐Solid Phase Change Materials Using Catalyst/Solvent‐Free Multi‐Component Reaction for Battery Thermal Management

**DOI:** 10.1002/advs.77157

**Published:** 2026-08-13

**Authors:** Changhui Liu, Guangyuan Liang, Yuanzheng Liu, Jiantang Gao, Zeyang Li, Lin Huang, Jiateng Zhao, Junbing Xiao, Xiaoxing Zhong, Jun (Joelle) Wang

**Affiliations:** ^1^ School of Low‐carbon Energy and Power Engineering China University of Mining and Technology Xuzhou Jiangsu China; ^2^ School of Energy and Power Engineering Changsha University of Science and Technology Changsha China; ^3^ Department of Chemistry Hong Kong Baptist University Kowloon Hong Kong

**Keywords:** catalyst‐free synthesis, flame retardancy, solid‐solid phase change materials, thermal management

## Abstract

Leakage and flammability are two critical safety issues limiting the practical application of organic phase change materials. Herein, a facile catalyst/solvent‐free multicomponent reaction involving alcohols/amines/thiols, dimethyl phosphite, and benzaldehyde was developed to simultaneously address these drawbacks. The terminal phosphite groups endow intrinsic flame retardancy, while intermolecular interactions enhanced by P═O bonds transform the material into a viscous‐to‐solid state above its melting point, eliminating leakage. Notably, the reaction proceeds smoothly with quantitative yield under fully catalyst‐free conditions as nucleophilicity increases from alcohols to thiols. Battery thermal management tests show the intrinsic flame‐retardant solid‐solid phase change materials extend the safe operation of lithium‐ion batteries by 140%. Cone calorimeter tests reveal a 14 s ignition delay, and 37.6%, 29.8%, and 27.3% reductions in average heat release rate, total heat release, and total oxygen consumption compared to raw materials, confirming excellent flame retardancy. The material exhibits a maximum latent heat of 133.87 J/g, ensuring favorable thermal energy storage performance.

## Graphical abstract

The phosphite group endows intrinsic flame retardancy, while the P = O bond enhances intermolecular interactions to realize solid‐solid phase transition. Battery thermal management tests show the materials extend the safe working time of lithium‐ion batteries by 200%. Cone calorimeter tests indicate a 14s delay in ignition time, and reductions of 37.6%, 29.8%, and 27.3% in peak heat release rate, total heat release, and total oxygen consumption, respectively, with a maximum latent heat of 133.87 J/g, achieving excellent flame retardancy and heat storage performance. This work synthesizes a series of intrinsic flame‐retardant solid‐solid PCMs and provides a green and efficient strategy for grafting phosphite groups onto organic PCMs.

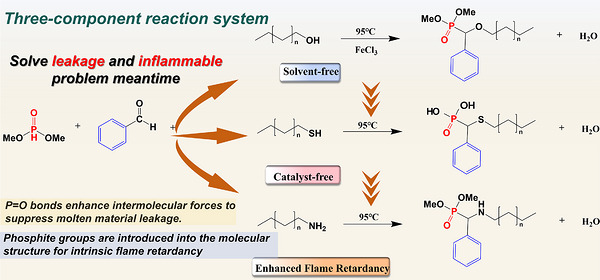

## 1 Introduction

Against the backdrop of advancing global energy structure transformation and deepening low‐carbon development strategies, the efficient storage and intelligent regulation of thermal energy have emerged as core technical bottlenecks [1, 2, 3, 4, 5]. Phase change materials (PCMs), leveraging their unique capability to absorb or release substantial latent heat during phase transition, offer an efficient solution for thermal energy storage and regulation, and have been widely applied in critical scenarios such as power battery thermal management [6, 7], industrial waste heat recovery [8, 9], and building temperature control [10, 11, 12]. Among these, solid‐liquid phase change materials (SLPCMs) have attracted extensive research attention owing to their high phase change latent heat and broad material selection scope [13, 14, 15, 16]. However, liquid leakage constitutes an inevitable challenge during their phase transition process [17, 18]. This issue not only impairs energy storage efficiency but also may trigger chain reactions such as equipment corrosion and potential safety hazards, thereby severely restricting their practical application [19, 20, 21, 22]. To address the leakage dilemma of SLPCMs, solid‐liquid phase change materials (SSPCMs) have been developed. These materials undergo only changes in crystal structure or molecular conformation during phase transition while maintaining a solid state throughout the process, thus fundamentally eliminating the risk of leakage [23, 24, 25, 26, 27].

To address the leakage issue of organic PCMs, existing studies predominantly employ techniques such as porous matrix encapsulation [28, 29, 30], polymer grafting [31, 32, 33, 34], and cross‐linking [35, 36, 37] polymerization to convert them into shape‐stabilized PCMs, among which chemical cross‐linking technology is the most widely applied. For instance, Wang et al. successfully constructed a polyethylene glycol (PEG)‐based SSPCM that is soluble and reprocessable at room temperature through dynamic ionic cross‐linking between Fe^3+^ and carboxylate groups. This material exhibits a maximum phase change latent heat of 97.6 J/g, excellent thermal stability, and nearly unchanged performance after multiple reprocessing cycles, enabling efficient and recyclable thermal energy storage [36]. Xia et al. incorporated graphene oxide into PEG molecular chains via self‐assembly and graft polymerization to construct a three‐dimensional layered structure, successfully developing a novel SSPCMs with high thermal conductivity, superior thermal cycling stability, and high phase change latent heat. The material achieves a melting enthalpy of 158.2 J/g, a phase change temperature of approximately, and a thermal conductivity enhancement of 41.4% compared to the pristine system, demonstrating outstanding thermal regulation capability and energy storage performance [38]. Yang et al. synthesized a cross‐linked photothermal SSPCMs using PEG as the phase change chain, triethanolamine as the cross‐linking agent, isophorone diisocyanate as the coupling agent, and p‐benzoquinone dioxime as the built‐in photothermal agent, with the cross‐linking density regulated by adjusting the NCO/OH molar ratio. The optimal sample exhibits a mechanical toughness of 166.38 MJ/m3, a phase change enthalpy of 93.2 J/g, a photothermal conversion efficiency of 87.48%, as well as excellent solvent resistance and reprocessability [39]. However, these methods have significant limitations. On the one hand, the preparation processes are complex and costly, and the organic solvents used in some preparation procedures are prone to causing secondary pollution, which is inconsistent with the concept of green production. Meanwhile, initiators and catalysts are usually required, and the reaction conditions are harsh, making it difficult to precisely regulate the phase change temperature and latent heat of the products.

At the same time, the synthesis strategy of new SSPCMs based on molecular design has attracted attention [40]. Researchers introduce groups such as hydrogen bond donors/acceptors into the molecular structure and use intermolecular forces to construct a stable three‐dimensional network structure, thereby achieving solid‐solid phase change transition. For example, Huang et al. adopted the prepolymer method: they used PEG 4000 as the soft segment, formed oxime‐urethane bonds between HDI and DMG, introduced Cu^2+^ to construct metal‐ligand bonds, and then formed a 3D network through PTOL crosslinking, finally preparing a polyurethane SSPCMs containing triple dynamic bonds. This material exhibits excellent shape stability, with a phase change enthalpy of 81.87∼86.67 J/g and a maximum tensile strength of 20.09 MPa. It also has photothermal conversion performance and can be reshaped and processed multiple times, which significantly improves the material's sustainability and practicality [41]. Zhu et al. prepared a material with high tensile strength, excellent toughness, healability and recyclability by coupling dynamic boroxane bonds with hydrogen bonds. After embedding oriented graphene nanosheets, the material achieved an oriented thermal conductivity of 3.639 W/(m·K) at a 5wt.% loading, enabling efficient solar thermal conversion, transfer and storage. It is suitable for the field of thermal energy storage [42]. Although such molecular design‐based synthetic strategies have demonstrated significant advantages in enhancing the performance of SSPCMs and provided an effective pathway for their functional expansion, the current technical system still has core drawbacks that urgently need to be addressed, mainly focusing on two dimensions: the lack of flame‐retardant properties and the complexity of preparation processes.

In terms of the flame retardant modification of PCMs, two main strategies are currently adopted: additive flame retardancy and intrinsic flame retardancy [43, 44]. The additive flame retardancy strategy achieves the flame retardant function by blending inorganic flame‐retardant such as magnesium hydroxide [45], aluminum hydroxide [46] or organic flame‐retardants such as intumescent flame retardants and phosphorus‐based flame retardants with PCMs [47, 48]. However, the addition of a large amount of flame‐retardant will significantly reduce the latent heat of phase change of the materials, and may also cause problems such as phase separation and decreased mechanical properties [49, 50]. The intrinsic flame‐retardant strategy achieves the synergistic optimization of flame‐retardant performance and phase‐change performance by introducing flame‐retardant elements such as phosphorus and nitrogen into the molecular structure of phase‐change materials [51, 52]. Wang et al. reacted vanillin with octadecylamine at 80°C to form a Schiff base, which was then reacted with diethyl phosphite to synthesize VOD, an intrinsic flame‐retardant PCMs integrating acid source, carbon source, and gas source. The resulting intrinsic flame‐retardant material exhibited a melting enthalpy of 59.4 J/g and stable performance after thermal cycling [53]. Li et al. investigated the two‐step synthesis of an intrinsic flame‐retardant PCM using octadecylamine, furfural, and diethyl phosphite as raw materials. This material possessed excellent flame retardancy and good thermal stability, and showed a favorable temperature control effect on lithium batteries under 3C discharge condition. However, all the aforementioned intrinsic flame‐retardant PCMs rely on multi‐step synthesis routes [54]. These routes not only increase the complexity of the preparation process and extend the production cycle, but also raise the production cost due to multi‐step operations, which limits their large‐scale industrial application. To address these issues, we have developed a novel preparation strategy for intrinsic flame‐retardant PCMs, enabling the one‐step synthesis of target products without the addition of any catalysts.

This study is dedicated to developing an intrinsically flame‐retardant SSPCM via a catalyst‐free, one‐step synthesis route, aiming to achieve the synergistic optimization of material properties through green preparation technology; using benzaldehyde (BA), dimethyl phosphite (DMP), and long‐chain alcohols amines thiols as raw materials a three‐component reaction system is constructed. By leveraging the synergistic autocatalytic effect of intermolecular hydrogen bonding P═O bond interaction and hydrophobic interaction the one‐step synthesis of the material is realized which avoids catalyst residues and simplifies the process flow. Regulating the carbon chain length of long‐chain raw materials enables precise tuning of the onset phase change temperature of the material within the range of −43.28 °C to 38.38 °C, thereby meeting the temperature control requirements of diverse application scenarios. Meanwhile flame‐retardant elements such as phosphorus and nitrogen are synchronously introduced into the molecular structure to construct an intrinsic flame‐retardant system that effectively addresses the safety hazards of flammability and leakage commonly existing in traditional PCMs. And adopting a solvent‐free “one‐pot” synthesis process, this technology features environmental friendliness and industrialization potential, providing a novel pathway for the low‐cost and large‐scale preparation of high‐performance, high‐safety SSPCMs.

## 2 Results and Discussion

### 2.1 Material Preparation

In this study, new PCMs were synthesized by a simple one‐pot method. First, DMP, BA and amine were added into a flask at a specific ratio. The mixture was stirred at 95°C until it became homogeneous, with a constant stirring speed of 400 rpm maintained throughout the reaction stage. The reaction process was terminated after a continuous reaction of 48 h. From the perspective of the reaction mechanism, under the combined action of high temperature, the carboxyl group of BA undergoes cleavage to form an electrophilic center. In contrast, due to the relatively small PKa values of the P‐H bond in DMP and the hydroxyl group of alcohol, H^+^ ions are more easily dissociated in the system, thereby forming a nucleophilic center [55, 56]. Driven by the electrostatic attraction between positive and negative charges, a dehydration condensation reaction occurs among the three components. After the reaction, the moisture in the product was removed through simple high‐temperature distillation. A series of preliminary experiments were performed to screen reaction conditions, and the optimal parameters, including reactant molar ratio, catalyst type and reaction time, were determined comprehensively based on product latent heat and phase‐change morphology. The 1:1:1 feeding ratio of dimethyl phosphite, benzaldehyde and long‐chain substrates was not selected arbitrarily. This proportion strictly conforms to the stoichiometric requirement of the three‐component dehydration condensation. Moreover, our experimental optimization further verified that the 1:1:1 ratio contributed to the highest reaction yield and optimal phase‐change performance, and the related results have been supplemented in the Supporting Information(Figures S1–S5, Tables S1 and S2).

**FIGURE 1:**
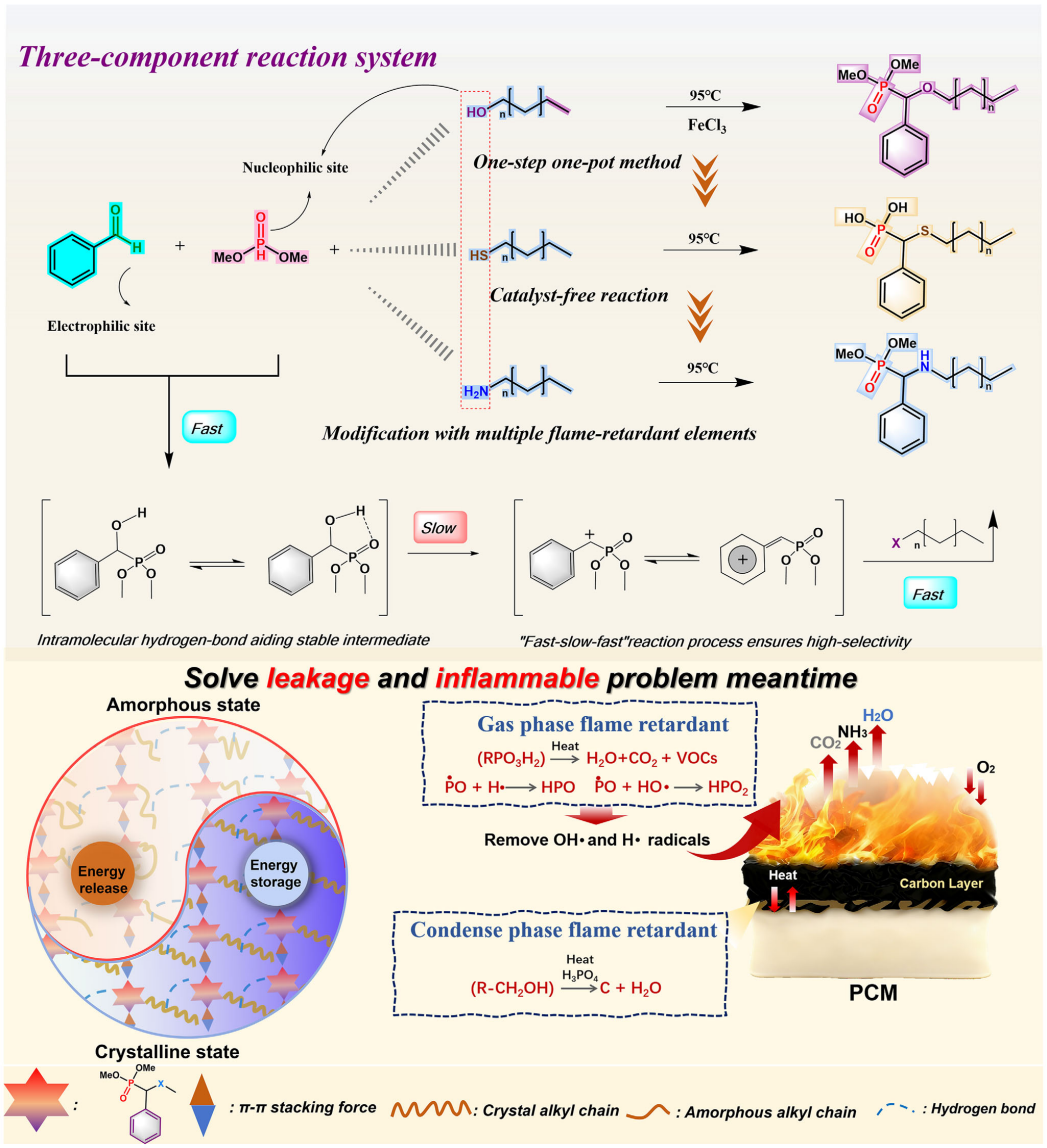
Schematic diagram of product's performance.

On the basis of the aforementioned research, we prepared SSPCMs via the reaction of BA and DMP with long‐chain thiols or long‐chain alcohol, respectively. Among these reactions, the three‐component reaction of aldehyde, ester, and alcohol has been reported in our previous work, and the detailed information of the samples is listed in Table 1 (the latent heat and onset phase change temperature of the samples are presented in Figures S6–S8 and Table S3). Figure 1 illustrates the synthetic route of the samples and their derivative variations. The three‐component reactions of BA, DMP with long‐chain amines, thiols, and alcohols were carried out via a one‐pot method under mild conditions. The reaction with alcohols requires FeCl_3_ as a catalyst, whereas reactions with thiols and amines proceed without catalysts. The thiol‐based samples show enhanced latent heat, and the amine‐based products exhibit improved flame retardancy due to nitrogen introduction [57].

**TABLE 1:** Sample information.

Sample	Raw material	Reaction ratio	Sample	Raw material	Reaction ratio
**SP1**	DMP、BA Tetradecanol	1: 1: 1	**SP6**	DMP、BA Octadecanethiol	1: 1: 1
**SP2**	DMP、BA Hexadecanol	1: 1: 1	**SP7**	DMP、BA Laurylamine	1: 1: 1
**SP3**	DMP、BA Octadecanol	1: 1: 1	**SP8**	DMP、BA Tetradecylamine	1: 1: 1
**SP4**	DMP、BA Tetradecanethiol	1: 1: 1	**SP9**	DMP、BA Hexadecylamine	1: 1: 1
**SP5**	DMP、BA Hexadecanethiol	1: 1: 1	**SP10**	DMP、BA Octadecylamine	1: 1: 1

The quantitative yield of amines and thiols under catalyst‐free conditions arises from the increasing nucleophilicity from alcohols to thiols. Thiols and amines possess higher nucleophilicity than alcohols, enabling spontaneous deprotonation and efficient nucleophilic attack without external catalysts [58]. Intermolecular hydrogen bonding and P = O interactions provide autocatalysis to stabilize transition states, lower energy barriers, and suppress side reactions. In contrast, alcohols show weak nucleophilicity and need FeCl_3_ as a Lewis acid catalyst for activation [40]. The high nucleophilicity of thiols and amines drives smooth and selective full conversion, affording quantitative yield under catalyst‐free conditions.

### 2.2 Characterization and Structural Analysis of Resultant Materials

**FIGURE 2:**
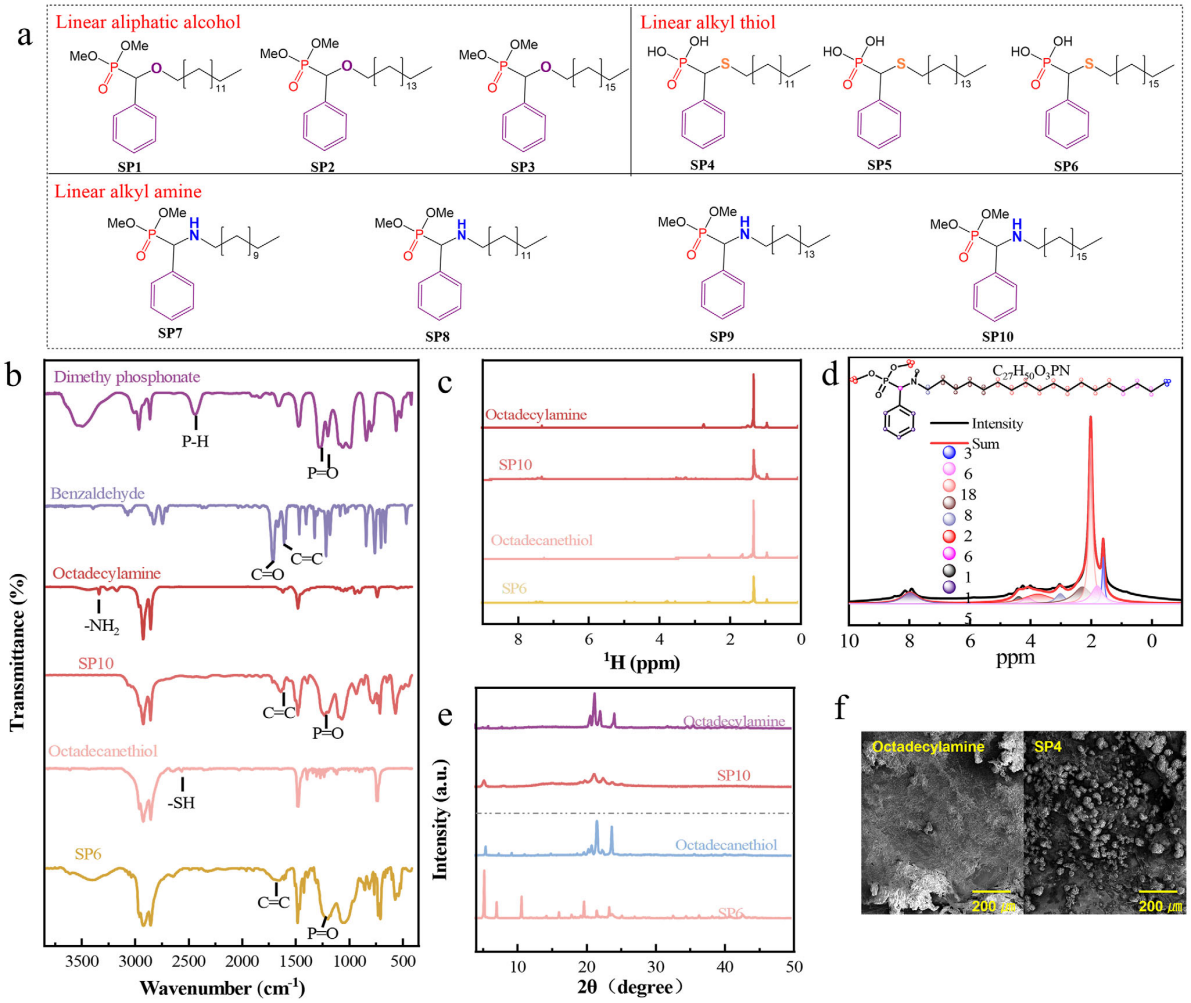
(a) Product Structure; (b) FTIR data analysis; (c) ^1^HNMR spectra of octadecanethiol, **SP6**, octadecanethiol, octadecylamine and **SP10**; (d) Solid‐state NMR analysis analysis of **SP10**.; (e) XRD patterns of octadecanethiol, **SP6**, octadecylamine and **SP10**; (f) SEM of octadecylamine and **SP10**.

As shown in Figure 2a, the ten prepared samples exhibit no significant structural differences. However, due to variations in the substituted functional groups, the product properties show obvious discrepancies. The products were thus classified into three groups (A, B, and C) based on the type of raw materials and subsequently subjected to characterization analysis. Fourier transform infrared spectroscopy(FTIR) can determine the formation of reaction products based on the appearance and disappearance of functional group characteristic bands. In this study, **SP6** and **SP10** were selected for characterization analysis, with their FTIR spectra presented in Figure 2b. Dimethyl phosphite exhibits P═O stretching vibration at 1250 cm^−1^ and C─O stretching vibration at 1040 cm^−1^. Benzaldehyde shows C═O stretching vibration around 1710 cm^−1^ and aromatic ring C═C stretching vibration near 1598 cm^−1^. The double band at 3300–3400 cm^−1^ corresponds to ─N─H stretching vibration, a core criterion for primary amines that distinguishes them from alkanes without N─H groups. Meanwhile, octadecanethiol is characterized by the ‐SH group absorption band at 2500 cm^−1^. From the FTIR spectra of each material, it can be seen that the characteristic bands of C═O stretching vibration and ─NH stretching vibration in **SP10** disappeared, while new absorption bands emerged at 1598 cm^−1^ and 1250 cm^−1^. This preliminary suggests that the three‐component raw materials underwent a reaction, and the resulting product is basically consistent with the predicted structure. The infrared characteristic bands of **SP6** also showed the same result: the ‐SH characteristic band in **SP6** disappeared, and C═C and P═O functional groups were successfully modified. Proton nuclear magnetic resonance spectroscopy (^1^H NMR) is an important technique for analyzing the molecular structure of organic compounds. As shown in Figure 2c, Compared with the starting materials octadecylamine and octadecanethiol, the products **SP6** and **SP10** exhibit characteristic signals of methyl protons in dimethyl phosphite and aromatic protons of the benzene ring, accompanied by the complete disappearance of sulfhydryl and amino signals from the raw materials, which fully confirms the successful three‐component modification of the starting materials. The results in Figure 2d further confirms the successful synthesis of the target product. Taking the solid‐state ^1^H NMR spectrum of **SP10** as an example, the fitting of the chemical shift peaks and integral ratios of hydrogen atoms is basically consistent with the theoretical number of hydrogen atoms. Further analysis of the **SP6** NMR spectra showed that all aromatic hydrogen signals of the benzene ring were clearly resolved. Meanwhile, characteristic hydroxyl hydrogen signals appeared in the spectra due to hydrolysis of the product (Figure S9). High Resolution Mass Spectrometry(HMRS) results further confirmed the successful synthesis of the target product (Figures S10 and S11). These results collectively verify the feasibility and effectiveness of the synthetic strategy adopted in this study.

Additionally, x‐ray Diffraction (XRD) analysis revealed obvious changes in both band intensity and number of **SP6** and **SP10** compared with the raw materials, indicating the formation of new crystal phases (Figure 2e). Scanning electron microscopy (SEM) results further confirmed the successful synthesis of the products (Figure 2f). A comparison of the microstructural changes between the products and raw materials before and after the reaction showed that octadecylamine exhibited a relatively loose flocculent and sheet‐like structure with loosely bound particles, high surface roughness, and irregular morphology. This structure indicates weak intermolecular forces among octadecylamine molecules, which exist as relatively independent molecular aggregates, and the intermolecular binding force is insufficient to form a stable dense structure. In contrast, **SP10** presented a large number of regular granular structures with uniform particle size, tight interparticle bonding, and a denser, more regular surface. This structure suggests that after the reaction, molecules interacted through stronger intermolecular forces, which significantly enhanced the cohesion and transformed the product from loose molecular aggregates into stable granular assemblies.

### 2.3 Thermal Physical Performance of PCMs Composites

**FIGURE 3:**
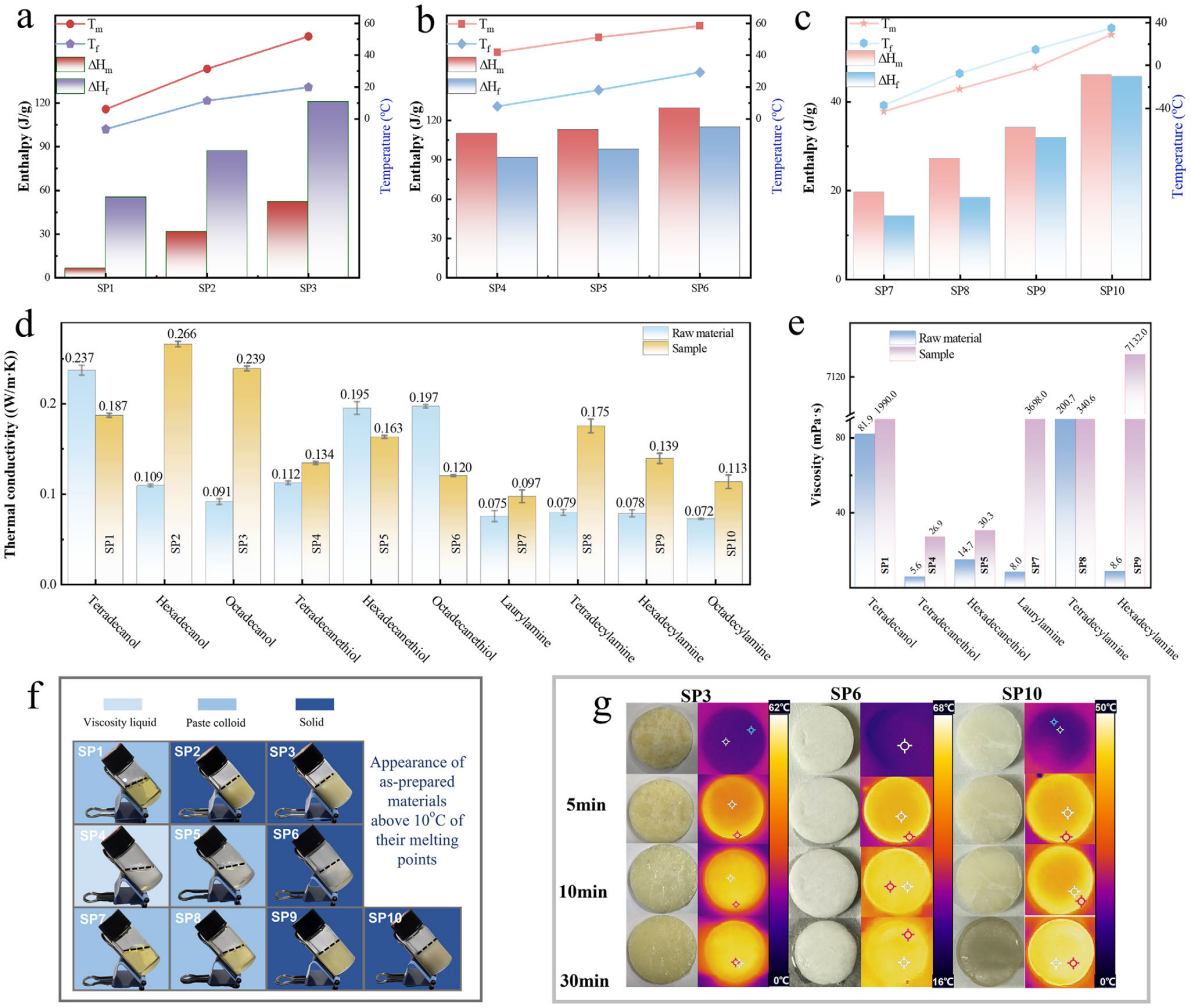
(a,b,c)The phase change latent heat and phase change temperatures of the samples; (d) The comparison of thermal conductivity between reactant raw materials and product; (e) Viscosity tests of liquid and paste‐like products; (f) The morphology of all products above the phase transition temperature; (d) The leakage tests for **SP3, SP6** and **SP10**.

The phase change properties of the series of products, including crystallization temperature, crystallization enthalpy, melting temperature, and melting enthalpy, were analyzed by Differential scanning calorimetry (DSC). As illustrated in Figure 3a,b,c, the reaction raw materials were divided into three groups (A, B, and C), and it can be clearly observed that both the phase change temperature and latent heat of the products increase with the increase in the relative molecular mass of the reaction raw materials. This is because as the relative molecular mass of the raw materials increases, the alkyl chains in the product molecules become longer and more ordered. Longer alkyl chains lead to stronger van der Waals forces and hydrophobic interactions between molecular chains, which significantly enhance the overall intermolecular interactions. As a result, greater thermal energy is required to disrupt the ordered structure and induce the phase transition, leading to a clear upward trend in the phase change temperature of the materials [59]. Among all samples, the maximum phase change latent heat reaches 133.87 J/g, demonstrating excellent thermal properties. Meanwhile, by regulating the amine, thiol, or alcohol raw materials reacted with BA and DMP, wide‐range regulation of the phase change temperature of product can be achieved. The onset phase change temperature ranges from −43.28°C to 38.38°C, which can meet the application requirements of various temperature control scenarios.

In addition to its excellent performance in latent heat, the product also shows improvement in thermal conductivity. As shown in Figure 3d, the thermal conductivity of the products in each group is higher than that of the corresponding raw materials, with the most significant improvement observed in Group A—all products show different degrees of increase compared with the raw materials. In Group C, the highest thermal conductivity of the product reaches 0.266 W/(m·K), which is 244% of that of the raw material.

The introduced aromatic rings and *π*–*π* stacking interactions effectively reduce phonon scattering and promote phonon transfer between molecular chains: aromatic rings provide a rigid molecular framework and delocalized *π*‐electron system that suppresses disordered molecular motion and decreases phonon scattering; *π*–*π* stacking forms a continuous ordered stacking structure to build efficient phonon transport pathways, while delocalized *π*‐electrons facilitate phonon and hot‐electron propagation; strong *π*–*π* interactions enhance intermolecular binding, reduce molecular gaps and voids, and avoid phonon scattering caused by structural defects [60, 61]. Compared with liquid or paste‐like products, solid products have lower intermolecular vibration freedom, which reduces phonon scattering and thus further improves thermal conductivity. In addition, the viscosity of the products shows an increasing trend with the increase in the relative molecular mass of the reaction raw materials (Figure 3e). The viscosity of all samples was measured at a temperature 10°C above their respective melting point peaks, and each group of samples also exhibits a trend of viscosity increasing with molecular weight. Among them, the viscosity of **SP9** reaches 7132.0 mPa·s, which is 830 times that of the raw material. When the molecular weight of the reaction raw materials further increases, the products exhibit solid‐solid phase change behavior. Figure 3f more intuitively demonstrates that all samples were imaged at a temperature 10°C above their respective melting point peaks. As the relative molecular mass of the reaction raw materials increases, the product state gradually transitions from a liquid to a gel, and finally forms a product with solid‐solid phase change behavior. Similar performance was observed in the leakage test (Figure 3g). The leakage of samples was monitored using an infrared camera; samples and raw materials were placed in an oven at a temperature 10°C higher than their melting point peaks. After 30 min, no obvious leakage was observed for **SP3** and **SP10**, indicating a distinct solid‐solid phase change behavior, whereas octadecylamine showed significant leakage after 10 min. **SP6** exhibited stronger high‐temperature resistance than the raw materials (shown in the Figure S12), confirming its excellent thermal stability. Other solid products also exhibit excellent thermal stability (Figure S13)

**FIGURE 4:**
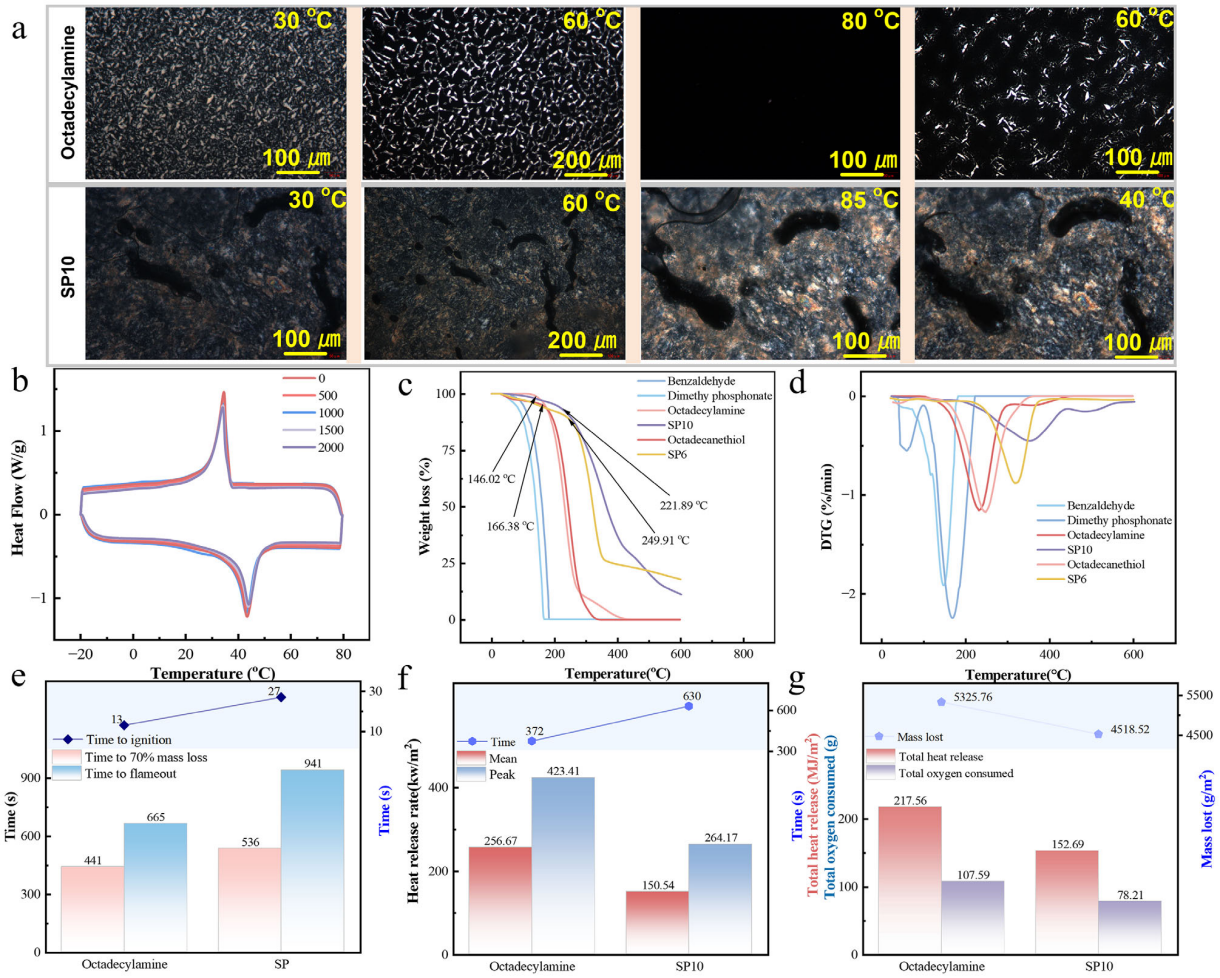
(a) The POM images of octadecylamine and **SP10** at different temperatures (b) **SP10** cycle diagram; (c) The TG diagram of **SP6, SP10**; (d) The DTG diagram of **SP6**, **SP10**; (e) Time required for ignition; (f) Heat release rate; (g) Oxygen consumed and mass loss.

Polarized optical microscopy (POM) characterization further validated the SSPCM nature of the products. As illustrated in Figure 4a, both octadecylamine and **SP10** exhibit distinct crystalline structures at room temperature, with significant differences in their crystal morphologies. This phenomenon further validates the effectiveness of the adopted synthesis strategy. With increasing test temperature, octadecylamine shows a reduction in crystal content near its phase transition temperature (approximately 60°C), indicating the initiation of melting. Owning to the rapid melting rate, octadecylamine has completely transformed into a liquid state when the temperature rises to 80°C. During the cooling process, octadecylamine re‐solidifies only near its phase transition temperature, demonstrating typical solid‐liquid phase change characteristics. In sharp contrast, **SP10** displays no obvious changes in crystal morphology even when heated to 20°C above its phase transition temperature, and maintains a solid state even at 85°C, exhibiting typical SSPCM behavior (the temperature‐varying POM test process is illustrated in Videoes S1 and S2). Furthermore, no significant alterations in its crystal morphology are observed after cooling, which confirms that **SP10** possesses excellent thermal stability.

Owing to the flame‐retardant modification of phosphorus element in **SP10**, coupled with the certain flame‐retardant property of nitrogen element in its long‐chain amine structure, **SP10** and phenolic resin amine were mixed at a mass ratio of 1:1 for cone calorimeter test. As shown in Figure 4e, the ignition time of the experimental group was more than twice that of the blank group, and the burning rate also decreased significantly. Specifically, the blank group reached the mass retention node of 70% at 441s, while this node was delayed by 95s in the experimental group, and the complete burning time of the experimental group was nearly 1.5 times that of the blank group. The above‐mentioned flame‐retardant advantages were also reflected in the heat release characteristics, as shown in Figure 4f. The average heat release rate of the experimental group was 150.54 kW/m^2^, accounting for only 58.6% of that of the blank group. Meanwhile, the peak heat release rate of the experimental group was 264.17 kW/m^2^, which was 47.6% lower than that of the blank group, and the occurrence time of peak heat release rate was delayed by 258s—this feature can provide more time for personnel evacuation in case of fire. In terms of overall combustion performance (Figure 4g), the experimental group not only exhibited less mass loss, but also had a total heat release of 152.69 kW/m^2^, equivalent to 70% of the blank group, and the oxygen consumption was reduced by nearly 30 g compared with the blank group. And significant differences were observed between the two groups of samples before and after combustion. The raw material group was completely burned, leaving only ashes. In contrast, the experimental group mixed with **SP10** retained its main structural integrity after combustion (shown in the Figure S17). This phenomenon is primarily attributed to the presence of phosphorus in **SP10**, which promotes the formation of a dense carbon layer during the combustion process.

In comparison with commercial flame‐retardant phase change materials in Table 2, our prepared sample achieves excellent fire resistance with no extra flame retardant added. It shows much lower PHRR and THR values than those modified with DOPO, PEPA and other common commercial flame retardants. Benefiting from the intrinsic phosphorus‐containing molecular structure, it avoids the drawbacks of high filler dosage and poor compatibility, and possesses more outstanding comprehensive fire safety and practical application value.

**TABLE 2:** Comparison of fire behavior with other flame‐retardant phase change materials.

Literatures	Ingredient	Types of added flame retardants(wt.%)	PHRR (kW/m^2^)	THR (MJ/m^2^)	Time to ignition(s)
This work	BA, DMP, Octadecylamine	None	264.14	152.69	27
[62]	PEG2000, EG, ER	APP(10%)	549.7	118	No report
[63]	PA, HDPE	APP + PER + MMA(25%)	569.19	88.8	26
[64]	Cu, HNTs, EP, CA, PMMA	DOPO	923	117	39
[44]	POCl_3_, hexadecanol	PEPA(20%)	1088	239	116
[65]	PA, EVA‐g‐M, EG	MA(25%)	696.5	226.9	No report

The phosphorus element in **SP10** and the nitrogen element in its long‐chain amine structure form a synergistic flame‐retardant effect, which enhances the flame‐retardant performance of the material by inhibiting combustion reactions, delaying heat release, and reducing the decomposition of combustibles. This flame‐retardant system has potential application value in fields such as building materials, electronic device packaging materials, and cable insulation layers. It can improve the fire safety level of related products by extending the safety time in the early stage of a fire and reducing the spread rate of the fire

### 2.4 Application

The products exhibit certain flame‐retardant properties due to the introduction of phosphorus. Moreover, cone calorimeter test results indicate that **SP10** possesses excellent flame‐retardant performance. Meanwhile, the strengthening effect of phosphorus and the synergistic interaction between polar and non‐polar components enable macromolecules to fully exert their latent heat advantages. Thus, **SP10** was selected for application experiments.

A comparison between the as‐prepared sample and recently reported solid‐solid phase change materials is summarized as follows. As shown in Table 3, the listed literatures mainly focus on conventional thermal energy storage, asphalt deicing, recyclable materials, and functional textiles, with phase transition temperatures ranging from sub‐zero to around 60°C and melting latent heat mostly between 37 and 120 J/g. Notably, none of the reported materials in the literatures possess flame‐retardant properties, which limits their application in scenarios with high safety requirements. In contrast, the material in this work not only achieves a high melting latent heat of 133.87 J/g at a suitable phase transition temperature of 38.38°C but also has intrinsic flame‐retardant performance due to the introduction of phosphorus. Specifically designed for battery thermal management, it demonstrates obvious advantages in thermal storage capacity, flame retardancy, and targeted application scenarios.

**TABLE 3:** Compared with other solid‐solid phase change materials.

Literatures	Ingredient	Latent heat of melting(J/g)	Phase change temperature (°C)	Application scenarios
This work	BA, DMP, Alcohols / Amines / Thiols	18.37 ∼ 133.87	−43.28 ∼ 38.38	Battery Thermal Management
[66]	MPEG, Vinyl unit	80 ∼ 110	36 ∼ 57	General thermal storage materials
[67]	PU, PTMEG2000, MDI	49.49	< ‐18	Asphalt pavements deicing and temperature control
[68]	CNCs, PEG	70 ∼ 100	35 ∼ 60	Solid‐solid phase change nanoparticles for thermal energy storage
[69]	PU, PTMEG, MDI, PEG	37.1	−5 ∼ 5	Asphalt pavements deicing
[70]	PEG, Silicone resins	50 ∼ 80	28 ∼ 40	Multifunctional textiles with electromagnetic interference shielding

**FIGURE 5:**
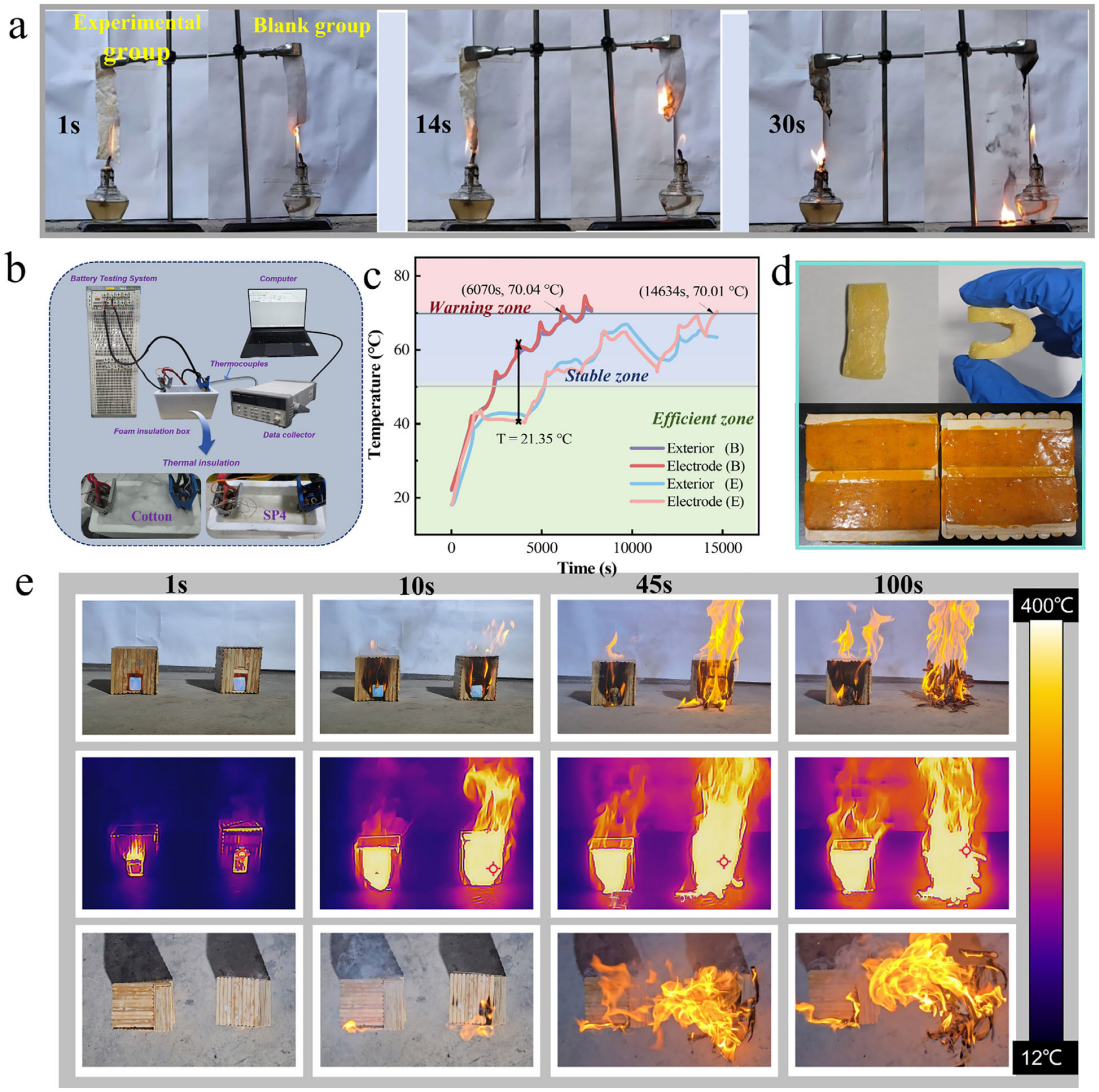
(a) Flame‐retardant performance evaluation of firefighting protective clothing; (b) Experimental setup for battery thermal management; (c) Temperature regulation performance in battery thermal management; (d) Properties of Flame‐Retardant Coatings; (e) Wood house combustion experiment.

In addition, during the sample preparation process for cone calorimeter tests, it was found that the mixture of **SP10** and phenolic resin at a 1:1 mass ratio exhibits excellent plasticity and strong adhesion after heating. As illustrated in Figure 5d, the mixture possesses good flexibility and maintains high adhesion even after heating‐cooling cycles, thus being selected as the flame‐retardant coating for wooden houses. Combustion test results as showed in Figure 5e. At 10s, heat spread rapidly in the blank group house, which started intense combustion with charring marks appearing at the lower right corner, while no obvious changes were observed on the top of the experimental group; at 45s, the fire spread to the entire blank group house, while the experimental group still showed no significant combustion signs, confirming the remarkable flame‐retardant effect of the coating; at 100s, the blank group house collapsed and was completely burned, while the main structure of the experimental group remained intact with only slight combustion marks on the top, and finally burned out at 456s. This experiment further verified the practical application feasibility of the product. Such PCMs with dual functions of energy storage and flame retardancy also have application potential in scenarios such as paper document fire protection and safe coating protection (the complete test process is provided in Video S4).

## 3 Conclusion

This study addresses the challenges of liquid leakage in SLPCMs and catalyst dependence in SSPCM by developing a catalyst‐free one‐step synthesis strategy. Using BA, DMP, and long‐chain alcohols/amines/thiols as raw materials, high‐performance intrinsic flame‐retardant SSPCMs with a three‐dimensional network structure were successfully prepared via the synergistic autocatalysis of intermolecular hydrogen bonding, P═O bonding, and long‐chain hydrophobic interactions. By regulating the carbon chain length of long‐chain raw materials, the onset phase change temperature of the materials can be tuned over a wide range from −43.28°C to 38.38°C, with a maximum phase change latent heat of 133.87 J/g. The materials exhibit excellent thermal stability, showing no significant performance degradation after 300 thermal cycles, which completely solves the liquid leakage and flammability issues of traditional PCMs. In practical applications, the materials can extend the safe operating time of power batteries by 1.4 times in thermal management systems and endow fire‐fighting suits, wooden materials, etc., with long‐lasting flame‐retardant properties. Meanwhile, featuring solvent‐free synthesis, low cost, and industrialization potential, this work provides a novel functional material solution for the fields of new energy thermal management and flame.

Furthermore, the as‐prepared solid‐solid phase change materials with high durability and stable phase transition behavior also exhibit promising potential in energy harvesting applications beyond thermal management and flame retardancy. As recently demonstrated in advanced tandem systems integrating PCM with thermoelectric generators, robust SSPCM with excellent stability and leakage‐proof characteristics are highly desirable for efficient cold energy storage and sustainable thermoelectric energy conversion [71]. In view of the increasing demand for robust and reliable solid‐solid PCMs in advanced energy utilization systems, the solvent‐free, catalyst‐free, and scalable synthetic strategy proposed in this work provides a feasible platform for designing multifunctional PCMs toward broader energy‐related applications.

## Author Contributions

**Guangyuan Liang**: writing – original draft, methodology, project administration, writing – review and editing. **Zeyang Li**: investigation. **Xiaoxing Zhong**: supervision, visualization. **Jun (Joelle) Wang**: supervision, funding acquisition, visualization, writing – review and editing, writing – original draft. **Yuanzheng Liu**: methodology, investigation. **Jiantang Gao**: investigation, methodology. **Jiateng Zhao**: validation. **Junbing Xiao**: methodology, supervision. **Lin Huang**: methodology, validation. **Changhui Liu**: conceptualization, supervision, funding acquisition, writing – review and editing, writing – original draft.

## Conflicts of Interest

The authors declare no conflicts of interest.

## Supporting information




**Supporting File 1**: advs77157‐sup‐0001‐SuppMat.docx.

**Supporting File 2**: advs77157‐sup‐0002‐S1.mp4.

**Supporting File 3**: advs77157‐sup‐0003‐S2.mp4.

**Supporting File 4**: advs77157‐sup‐0004‐S3.mp4.

**Supporting File 5**: advs77157‐sup‐0005‐S4.mp4.

## Data Availability

The data that support the findings of this study are available from the corresponding author upon reasonable request.
