# Supplementary material for: Hydrogen‐Bond Enabled Phosphite Based Intrinsic Flame‐Retardant Solid‐Solid Phase Change Materials Using Catalyst/Solvent‐Free Multi‐Component Reaction for Battery Thermal Management
Source: Adv Sci (Weinh). 2026 Aug 13;13(61):e77157. doi: 10.1002/advs.77157 (PMC13472106; doi:10.1002/advs.77157)
Supplement: Supplementary file 1 [file ADVS-13-e77157-s003.docx]

**Hydrogen-bond Enabled Phosphite Based Intrinsic Flame-retardant Solid-solid PhaseChange Materials Using Catalyst/solvent-free Multi-component Reaction for BatteryThermal Management**

*Changhui Liu^a,^*, Guangyuan Liang^a^, Yuanzheng Liu^a^, Jiantang Gao^a^, Zeyang Li^a^, Lin Huang^a^, Jiateng Zhao^a^, Junbing Xiao^b,^*, Xiaoxing Zhong^a^,* *Jun (Joelle) Wang^c,^**

^a^*School of Low-carbon Energy and Power Engineering, China University of Mining and Technology, 221116, Xuzhou, China.*

*E-mail:* *xjb1th@163.com*

^b^*School of Energy and Power Engineering, Changsha University of Science and Technology, 410114, Changsha, China*

*Email: [liuch915@cumt.edu.cn](mailto:liuch915@cumt.edu.cn)*

*^c^Department of Chemistry, Hong Kong Baptist University, Kowloon, Hong Kong.*

*E-mail: junwang@hkbu.edu.hk*

**Content**

*[Fig S1 The influence of reaction time on the enthalpies of reaction products of aldehydes, esters, and amines](#_Toc229538133)*	[3](#_Toc229538133)

*[Fig S2 The influence of reaction time on the thermogravimetry of of reaction products of aldehydes, esters, and amines](#_Toc229538134)*	[4](#_Toc229538134)

*[Fig S3 The influence of catalyst on the thermogravimetry of of reaction products of aldehydes, esters, and amines](#_Toc229538135)*	[5](#_Toc229538135)

*[Fig S4 The influence of catalyst addition on product leakage](#_Toc229538136)*	[6](#_Toc229538136)

*[Fig S5 The influence of catalysts on the leakage of aldehyde, ester, and thiol products](#_Toc229538137)*	[7](#_Toc229538137)

*[Table S1 Adjust the optimal reaction ratio of aldehyde, ester and alcohol.](#_Toc229538138)*	[8](#_Toc229538138)

*[Table S2](#_Toc229538139)* *[Adjust the optimum reaction temperature.](#_Toc229538139)*	[9](#_Toc229538139)

*[Fig S6 The DSC Curve of the Product from BA, DMP and Long-Chain Alcohol](#_Toc229538140)*	[10](#_Toc229538140)

*[Fig S7 The DSC Curve of the Product from BA, DMP and Long-Chain thiol](#_Toc229538141)*	[11](#_Toc229538141)

*[Fig S8 The DSC Curve of the Product from BA, DMP and Long-Chain thiol .](#_Toc229538142)*	[12](#_Toc229538142)

*[Table S3 Latent heat and phase change temperature of the sample.](#_Toc229538143)*	[13](#_Toc229538143)

*[Fig S9](#_Toc229538144) ^[1](#_Toc229538144)^[H NMR analysis analysis of SP6](#_Toc229538144)*	[14](#_Toc229538144)

*[Fig S10 HRMS (ESI) of SP6](#_Toc229538145)*	[15](#_Toc229538145)

*[Fig S11 HRMS (ESI) of SP10](#_Toc229538146)*	[16](#_Toc229538146)

*[Fig S12 Leakage Test of SP6 and its raw material](#_Toc229538147)*	[17](#_Toc229538147)

*[Fig S13 Leakage Test of Other Solid Samples](#_Toc229538148)*	[18](#_Toc229538148)

*[Fig S14 FTIR spectra of SP10 after cycling test](#_Toc229538149)*	[19](#_Toc229538149)

*[Fig S15 XRD spectra of SP10 after cycling test.](#_Toc229538150)*	[20](#_Toc229538150)

*[Fig S16 TGA of SP10 after cyclic treatment.](#_Toc229538151)*	[21](#_Toc229538151)

*[Fig S17 Comparison of octadecylamine, SP10 and phenolic resin mixed samples before and after combustion.](#_Toc229538152)*	[22](#_Toc229538152)

*[Table S4 Battery parameters.](#_Toc229538153)*	[23](#_Toc229538153)

# *Fig S1 The influence of reaction time on the enthalpies of reaction products of aldehydes, esters, and amines*

The purity of the product increases with the extension of reaction time, and the DSC characteristic peaks gradually become single and uniform.

# *Fig S2 The influence of reaction time on the thermogravimetry of of reaction products of aldehydes, esters, and amines*

With the improvement of product purity, the thermal decomposition temperature of the sample is significantly increased.

# *Fig S3 The influence of catalyst on the thermogravimetry of of reaction products of aldehydes, esters, and amines*

Various catalysts show negligible effects on the three-component reaction system of benzaldehyde, dimethyl phosphite and octadecylamine. DSC analysis reveals that all groups of samples display identical peak shapes and thermal transition features, which verifies the insignificant influence of catalyst types.

# *Fig S4 The influence of catalyst addition on product leakage*

**5min**

**20min**

**15min**

**10min**

**30min**

**None**

**10% FeCl_3_**

**10% AlCl_3_**

**10% CuCl_2_**

**Test temperature：68℃**

The introduction of different catalysts cannot effectively improve the anti-leakage ability of the prepared samples. Catalyst variation exerts only a negligible effect on optimizing the solid-state stability and leakage resistance of the target products.

# *Fig S5 The influence of catalysts on the leakage of aldehyde, ester, and thiol products*

The introduction of different catalyst types fails to enhance the leakage resistance of thiol-based products, and changing catalytic conditions presents only a limited effect on improving their solid-state stability.

# *Table S1 Adjust the optimal reaction ratio of aldehyde, ester and alcohol.*

| The ratio of ester, aldehyde and alcohol | Coagulation temperature℃ | Enthalpy of Coagulation J/g | Melting temperature℃ | Enthalpy of melting J/g |
| --- | --- | --- | --- | --- |
| 1：1：1 | 19.88 | 120.75 | 51.86 | 133.87 |
| 1：1.1：1 | 23.40 | 85.28 | 40.80 | 109.21 |
| 1：1.2：1 | 23.75 | 71.94 | 48.94 | 74.47 |
| 1：1.3：1 | 23.22 | 57.96 | 40.64 | 110.82 |
| 1：1.4：1 | 20.02 | 55.77 | 47.73 | 99.75 |
| 1：1.6：1 | 19.59 | 71.87 | 38.46 | 82.47 |
| 1.1：1：1 | 24.31 | 83.51 | 52.00 | 113.45 |
| 1.2：1：1 | 25.02 | 66.24 | 53.89 | 55.54 |
| 1.4：1：1 | 25.03 | 66.98 | 51.30 | 64.28 |
| 1.5：1：1 | 23.76 | 63.47 | 47.18 | 61.28 |
| 1.6：1：1 | 16.55 | 76.46 | 43.39 | 93.79 |
| 1.2：1.3：1 | 23.24 | 55.99 | 51.56 | 63.21 |
| 1.3：1.2：1 | 24.11 | 69.93 | 51.23 | 83.21 |
| 1.2：1.1：1 | 23.91 | 78.46 | 43.23 | 56.68 |
| 1.1：1.2：1 | 21.67 | 67.09 | 49.48 | 84.82 |
| 1.1：1.1：1 | 24.39 | 82.97 | 54.49 | 95.02 |
| 1.2：1.2：1 | 23.65 | 67.06 | 52.66 | 80.26 |
| 1.3：1.3：1 | 23.67 | 71.86 | 52.66 | 82.52 |

As shown in the table, the molar ratio of 1:1:1 exhibits the optimal comprehensive thermal performance among all proportion groups. Under this ratio, the sample delivers the highest melting enthalpy and a satisfactory freezing enthalpy , along with appropriate melting and solidification temperatures. By contrast, adjusting the proportion of aldehyde or ester, and adopting other compound ratios, generally leads to an obvious reduction in phase change enthalpy and unstable phase transition temperatures. Therefore, the 1:1:1 molar ratio is confirmed as the most favorable formulation for preparing the target solid–solid phase change materials.

# *Table S2* *Adjust the optimum reaction temperature.*

| reaction temperature℃ | Coagulation temperature℃ | Enthalpy of Coagulation J/g | Melting temperature℃ | Enthalpy of melting J/g |
| --- | --- | --- | --- | --- |
| 80 | 25.41 | 102.47 | 41.00 | 70.42 |
| 85 | 22.01 | 84.20 | 56.99 | 96.45 |
| 90 | 26.69 | 81.86 | 38.24 | 84.60 |
| 95 | 19.88 | 120.75 | 51.86 | 133.87 |
| 100 | 23.48 | 90.47 | 52.06 | 100.75 |
| 105 | 22.50 | 88.01 | 53.46 | 101.34 |
| 110 | 23.74 | 99.04 | 51.68 | 110.12 |

It can be seen from the data that the reaction temperature significantly affects the phase-change thermal properties of the products. Among all tested temperature conditions, the sample synthesized at 95 °C presents the maximum melting enthalpy and solidification enthalpy. In comparison, either lower or higher reaction temperatures cause an obvious decrease in phase-change enthalpy and unsatisfactory thermal performance. Consequently, 95°C is determined as the optimal reaction temperature for this system.

# *Fig S6 The DSC Curve of the Product from BA, DMP and Long-Chain Alcohol*

# *Fig S7 The DSC Curve of the Product from BA, DMP and Long-Chain thiol*

# *Fig S8 The DSC Curve of the Product from BA, DMP and Long-Chain thiol .*

# *Table S3 Latent heat and phase change temperature of the sample.*

| specimen | Onset Condensation temperature℃ | | Enthalpy of condensation J/g | Onset Melting temperature℃ | Enthalpy of melting J/g |
| --- | --- | --- | --- | --- | --- |
| SP1 | -3.51 | 55.1 | | -13.70 | 51.28 |
| SP2 | 17.66 | 86.92 | | 7.77 | 95.77 |
| SP3 | 26.92 | 120.75 | | 28.21 | 133.87 |
| SP4 | 10.19 | 91.381 | | 26.28 | 109.79 |
| SP5 | 22.26 | 87.797 | | 38.38 | 112.83 |
| SP6 | 35.38 | 76.75 | | 46.51 | 129.23 |
| SP7 | -37.66 | 14.251 | | -43.28 | 19.61 |
| SP8 | -7.79 | 18.365 | | -22.37 | 27.178 |
| SP9 | 14.55 | 31.14 | | -2.31 | 34.36 |
| SP10 | 34.68 | 45.657 | | 28.61 | 46.035 |

# *Fig S9 ^1^H NMR analysis analysis of SP6*

Characteristic peaks of methyl groups in the DMP structure and aromatic hydrogen signals of the benzene ring were clearly observed in the spectra, confirming the smooth progress of the three-component reaction and the successful preparation of the target product. Nevertheless, the thiol-participated phosphonate products are prone to hydrolysis conversion from P–OCH₃ to P–OH. Fundamentally, the central phosphorus atom inherently possesses strong positive electropositivity. The introduction of sulfur atoms in the molecule further enhances the electron-deficient degree of phosphorus through the electron-withdrawing inductive effect and weakens the P–OCH₃ bond. Meanwhile, methoxy groups serve as excellent leaving groups. In addition, the octadecyl mercaptan system tends to retain trace acidic impurities, and the products readily absorb moisture from ambient air. Under the combined action of acid catalysis and nucleophilic attack of water molecules, the methoxy groups in the molecular structure are easily substituted by hydroxyl groups, thereby inducing the hydrolysis process.

# *Fig S10 HRMS (ESI) of SP6*

HRMS (ESI): calcd for C_25_H_45_O_3_PS^+^ [M+H] ^+^: 457.28998; found:457.28702

# *Fig S11 HRMS (ESI) of SP10*

HRMS (ESI): calcd for C_27_H_50_O_3_PN^+^ [M+H] ^+^: 468.36011; found:468.35901

# *Fig S12 Leakage Test of SP6 and its raw material*

**0min**

**5min**

**10min**

**30min**

**Octadecanethiol**

**SP6**

The tests were conducted at temperatures 10 °C above their respective melting points, with the testing temperature for the product set higher than that for the raw materials. It can be clearly observed that SP6 exhibits significantly superior anti-leakage performance at the same time point.

# *Fig S13 Leakage Test of Other Solid Samples*

All samples exhibited excellent thermal stability and maintained no leakage after 10 minutes when exposed to temperatures above their respective melting points. Among them, SP2 and SP9 showed no leakage even after 30 minutes under the same temperature condition.

# *Fig S14 FTIR spectra of SP10 after cycling test*

No obvious differences are observed in the FTIR spectra of SP10 before and after cyclic testing, indicating that the chemical structure of the sample remains well maintained after long-term cycling.

# *Fig S15 XRD spectra of SP10 after cycling test.*

No obvious differences are observed in the XRD patterns of SP10 before and after cyclic testing, demonstrating that the crystalline structure of the sample remains intact and stable after long-term cycling.

# *Fig S16 TGA of SP10 after cyclic treatment.*

TGA analysis shows that the cycled sample possesses a higher thermal decomposition temperature than the fresh counterpart. After cyclic treatment, the trace low-molecular volatile components are gradually eliminated, which effectively suppresses the volatilization behavior and further improves the overall thermal stability of the material.

# *Fig S17 Comparison of octadecylamine, SP10 and phenolic resin mixed samples before and after combustion.*

Significant differences were observed between the two groups of samples before and after combustion. The raw material group was completely burned, leaving only ashes. In contrast, the experimental group mixed with SP10 retained its main structural integrity after combustion. This phenomenon is primarily attributed to the presence of phosphorus in SP10, which promotes the formation of a dense carbon layer during the combustion process.

# *Table S4 Battery parameters.*

|  | **Battery type** | **Battery model** | **Nominal voltage** | **Rate of discharge** | **Diameter** | **Length** |
| --- | --- | --- | --- | --- | --- | --- |
|  | LiFePO_4_ | 47145（20Ah) | 3.2V | 10-20C | 47mm | 145mm |
